# Development of a lateral flow test for the rapid detection of *Avibacterium paragallinarum* in chickens suspected of having infectious coryza

**DOI:** 10.1186/s12917-018-1729-0

**Published:** 2018-12-19

**Authors:** Sandra Morales Ruiz, Jorge Bendezu, Ricardo Choque Guevara, Ricardo Montesinos, David Requena, Luz Choque Moreau, Ángela Montalván Ávalos, Manolo Fernández-Díaz

**Affiliations:** 1 Laboratorios de Investigación y Desarrollo, FARVET SAC, Carretera Panamericana Sur Nº766 Km 198.5, Chincha Alta, 11702 Ica Peru; 2 Laboratorio de Bioinformática y Biología Molecular, Laboratorio de Investigación y Desarrollo, Universidad Peruana Cayetano Heredia, Av. Honorio Delgado 430 San Martín de Porres, Lima, Lima Peru; 3 FARVET SPF SAC, Carretera Panamerica Sur Nº766 Km 198.5, Chincha Alta, 11702 Ica Peru

**Keywords:** *Avibacterium paragallinarum*, Infectious coryza, Lateral flow test (LFT), Monoclonal antibody, TonB-dependent transporter (TBDT)

## Abstract

**Background:**

Infectious coryza (IC) is an acute respiratory disease of growing chickens and layers caused by *Avibacterium paragallinarum*. The development of tools that allow rapid pathogen detection is necessary in order to avoid disease dissemination and economic losses in poultry. An *Av. paragallinarum*-specific Ma-4 epitope of the TonB-dependent transporter (TBDT) was selected using bioinformatic tools in order to immunize a BalbC mouse and to produce monoclonal antibodies to be used in a lateral flow test (LFT) developed for *Av. paragallinarum* detection in chicken nasal mucus samples.

**Results:**

The 1G7G8 monoclonal antibody was able to detect TBDT in *Av. paragallinarum* cultures (serogroups: A, B and C) by Western blot and indirect ELISA assay. Consequently, we developed a self-pairing prototype LFT. The limit of detection of the prototype LFT using *Av. paragallinarum* cultures was 1 × 10^4^ colony-forming units (CFU)/mL. Thirty-five nasal mucus samples from chickens suspected of having infectious coryza were evaluated for the LFT detection capacity and compared with bacterial isolation (B.I) and polymerase chain reaction (PCR). Comparative indicators such as sensitivity (Se), specificity (Sp), positive predictive value (PPV), negative predictive values (NPV) and the kappa index (K) were obtained. The values were 100.0% Se, 50% Sp, 65.4% PPV, 100% NPV, and 0.49 K and 83.9% Se, 100% Sp, 100% PPV, 44.4% NPV, and 0.54 K for the comparison of the LFT with B.I and PCR, respectively. Additionally, the LFT allowed the detection of *Av. paragallinarum* from coinfection cases of *Av. paragallinarum* with *Gallibacterium anatis*.

**Conclusions:**

The results indicate that the self-pairing prototype LFT is suitable for the detection of TBDT in *Av. paragallinarum* cultures as well as in field samples such as nasal mucus from *Av. paragallinarum-*infected chickens. Therefore, this prototype LFT could be considered a rapid and promising tool to be used in farm conditions for *Av. paragallinarum* diagnosis.

## Background

Infectious coryza (IC) is caused by *Avibacterium paragallinarum* (*Av. paragallinarum*), a gram-negative bacteria previously called *Haemophilus paragallinarum* [1]. IC is an acute respiratory disease of growing chickens and layers and is associated with reduced egg production in laying flocks and delay in growth due to decreased food and water consumption in young chickens [2]. The most common clinical signs are serous or mucous nasal exudates, sneezing, swelling of infraorbital sinuses, facial edema and conjunctivitis [2]. All these clinical signs caused by *Av. paragallinarum* have been associated with economic losses in the poultry industry [2] that highlight the necessity of developing reliable tools for *Av. paragallinarum* detection.

Previous studies emphasized the use of PCR methods in comparison with bacterial isolation for the detection of *Av. paragallinarum*. The difficulties and challenges of *Av. paragallinarum* identification are well known using the latter method, which is a time-consuming process [3–6]. Moreover, the use of PCR-based methods is less time-consuming, but the methods require well-trained personnel and sophisticated infrastructure in laboratories. On the other hand, the use of a lateral flow test for detection of *Av. paragallinarum* is an alternative method that is not demanding and is an easily performable task. This method requires the identification of a specific epitope in the target protein that is detected through the use of a monoclonal antibody.

TonB-dependent transporters (TBDTs) are membrane proteins that have high affinity for iron, vitamin B_12_, siderophores and carbohydrates, which are important for bacteria. Iron is the main substrate for TBDTs, and it participates in many bacterial metabolic processes [7].

These TBDT proteins have been identified inside outer membrane vesicles (OMVs) in culture supernatants of members of Pasteurellaceae, such as *Pasteurella multocida* [8]. The content of these OMVs has been previously associated with extracellular virulence factors released from bacteria that can damage host tissue [9]. These OMVs were also identified in *Av. paragallinarum* cultures, but TBDT presence inside these vesicles is still uncertain in culture supernatants [9].

The potential extracellular presence of TBDT makes it a promising candidate for *Av. paragallinarum* identification due to its simple detection by monoclonal antibodies. The application of monoclonal antibodies in previous works with *Av. paragallinarum* has been limited to serotyping [10, 11], strain-vaccine differentiation [12], inhibition of hemagglutination and vaccine development [13, 14]. This study will take advantage of the specificity that a monoclonal antibody provides for the identification of *Av. paragallinarum* through the development of a lateral flow assay.

## Results

### Monoclonal antibody characterization

Two hybridoma clones, 1G7G8 and 3A3D8, were selected based on their reactivity against the peptide (Ma-4); 1G7G8 and 3A3D8 produced monoclonal antibodies, which were characterized as IgG1 (k) and IgG2b (k) isotypes, respectively. The antibody titer was 4.1 × 10^− 4^ for each hybridoma culture.

### Identification of recombinant TBDT using the 1G7G8 and 3A3D8 monoclonal antibodies in a Western blot

**Fig. 1:**
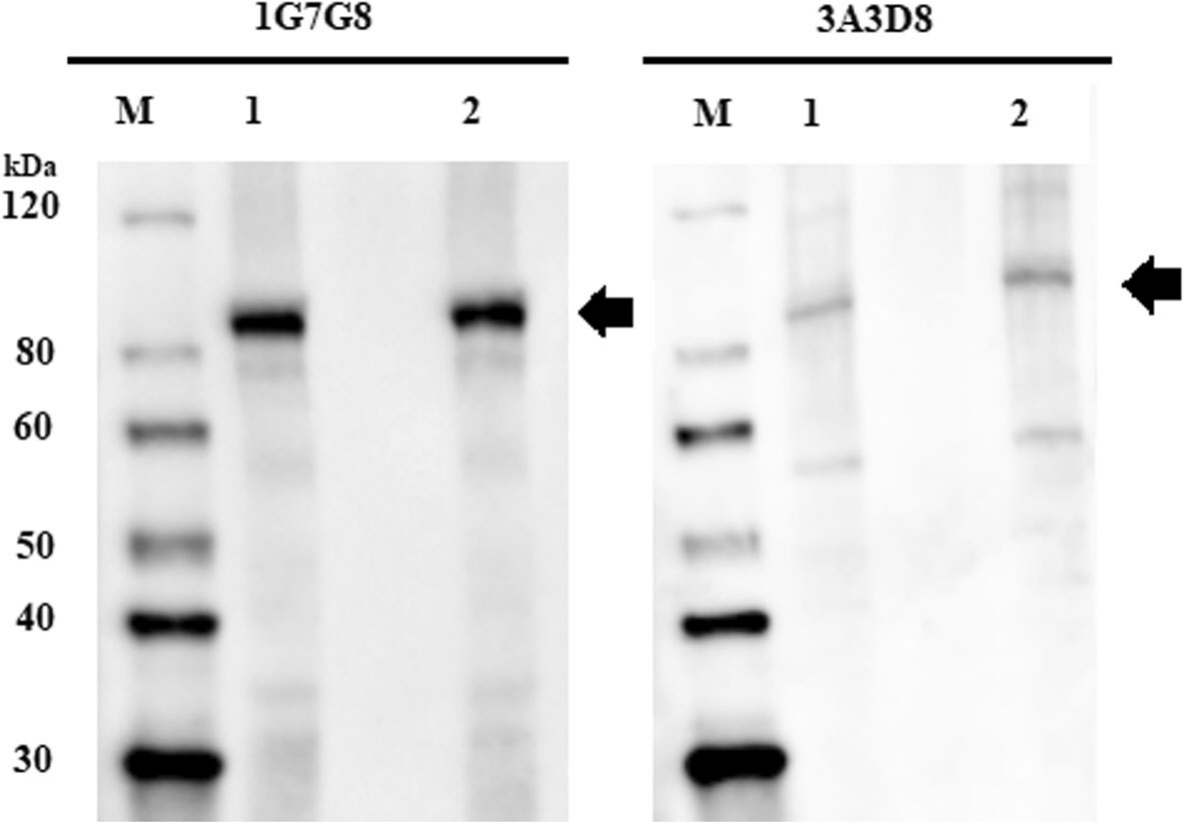
Identification of recombinant TBDT protein by Western blot assay using 1G7G8 and 3A3D8 antibodies. M: Molecular weight marker (30–120 kDa); Lane 1 and 2: recombinant TBDT (500 ng). Black arrows indicate the reactive band in each lane

Five hundred nanograms of recombinant protein were used for Western blot using purified monoclonal antibodies from 1G7G8 and 3A3D8 hybridoma clones. We obtained a unique reactive band of ⁓87 kDa corresponding to recombinant TBDT, demonstrating that the recombinant protein was recognized by 1G7G8 and 3A3D8 (Fig. 1).

### Identification of TBDT in *Av. paragallinarum* cultures (serogroups a, B and C) using the 1G7G8 and 3A3D8 monoclonal antibodies in a Western blot

**Fig. 2:**
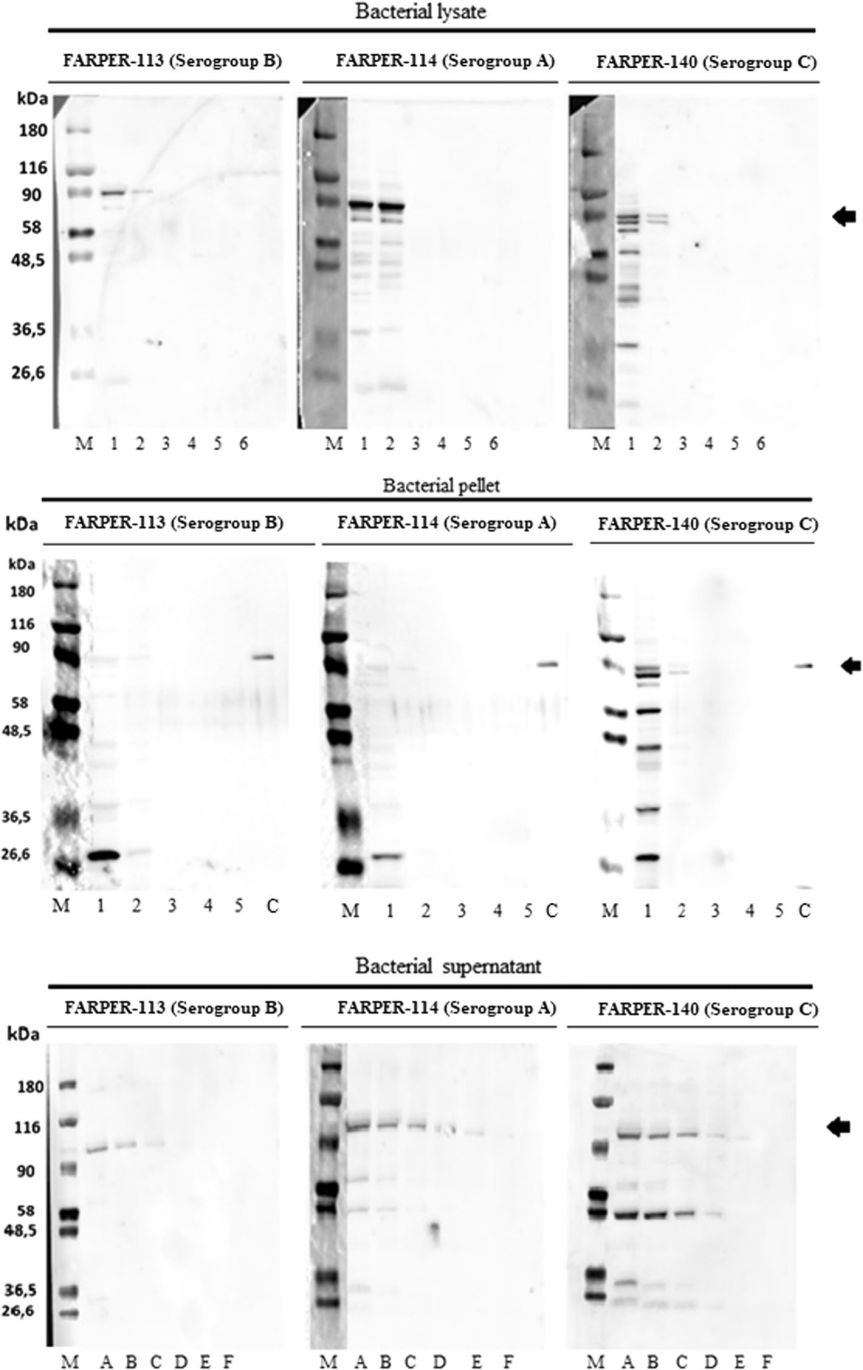
Identification of TBDT in *Avibacterium paragallinarum* cultures using the 1G7G8 monoclonal antibody in Western blot. *Avibacterium paragallinarum* cultures: FARPER-113 (serogroup B), FARPER-114 (serogroup A) and FARPER-140 (serogroup C). Lane M: Molecular weight marker (26.6–180 kDa). For bacterial lysate concentrations: lane 1 (500 μg/mL), lane 2 (50 μg/mL), lane 3 (5 μg/mL), lane 4 (0.5 μg mL), lane 5 (0.05 μg/mL), and lane 6 (0.005 μg/mL). For bacterial pellet: lane 1 (500 μg/mL), lane 2 (50 μg/mL), lane 3 (5 μg/mL), lane 4 (0.5 μg mL), lane 5 (0.05 μg/mL), and lane C (recombinant TBDT). For bacterial supernatant concentrations: (**a**) (undiluted supernatant), (**b**) (diluted 1:2), (**c)** (diluted 1:4), (**d**) (diluted 1:8), (**e)** (diluted 1:16), and (**f**) (BHI broth). Black arrows indicate the specific reactive band in each lane

The 1G7G8 and 3D3A8 monoclonal antibody reactivity was evaluated using bacterial lysates, pellets and supernatants from *Av. paragallinarum* cultures. Using the 1G7G8 antibody, in transfer protein membranes from bacterial lysates, we detected specific bands (~90 kDa) for serogroup B at 500 and 50 μg/mL, whereas for serogroup A, we detected specific stronger bands (~90 kDa) at the same concentrations. However, for serogroup C, multiple bands were observed at 500 μg/mL, and only two bands had similar molecular weight (~90 kDa) at 50 μg/mL. Regarding transfer protein membranes from the bacterial supernatant, we detected a specific band slightly above 90 kDa for serogroups B and A until dilutions of 1:4 and 1:16 were achieved, respectively. However, nonspecific weak bands were also observed in serogroup A at the lowest dilutions. For serogroup C, we observed two stronger bands of different molecular weights of ~90 and ~48.5 kDa until a dilution of 1:8 was reached (Fig. 2). For pellet samples, no bands were detected for serogroups A and B. However, we observed non-specific bands including a band of ~90 kDa at 500 μg/mL and a weak band at 50 μg/mL for serogroup C. On the other hand, no bands were detected for any samples using the 3A3D8 antibody in Western blot (data not shown). Therefore, this antibody was not used for further evaluations.

### Evaluation of the 1G7G8 monoclonal antibody reactivity against related and non-related bird bacterial cultures in Western blot

1G7G8 reactivity was evaluated against other bacteria, such as *Gallibacterium anatis*, *Pasteurella multocida, Ornithobacterium rhinotracheale* and *Bortedella bronchiseptica*, using bacterial pellets, lysates and supernatants in Western blot.

For supernatant samples, no bands were detected for *G. anatis*, *P. multocida*, *O. rhinotracheale* or *B. bronchiseptica*. Furthermore, for lysate samples, a weak, nonspecific band of low molecular weight was detected for *B. bronchiseptica* at 500 and 50 μg/mL, whereas no bands were detected for other bacteria.

**Fig. 3:**
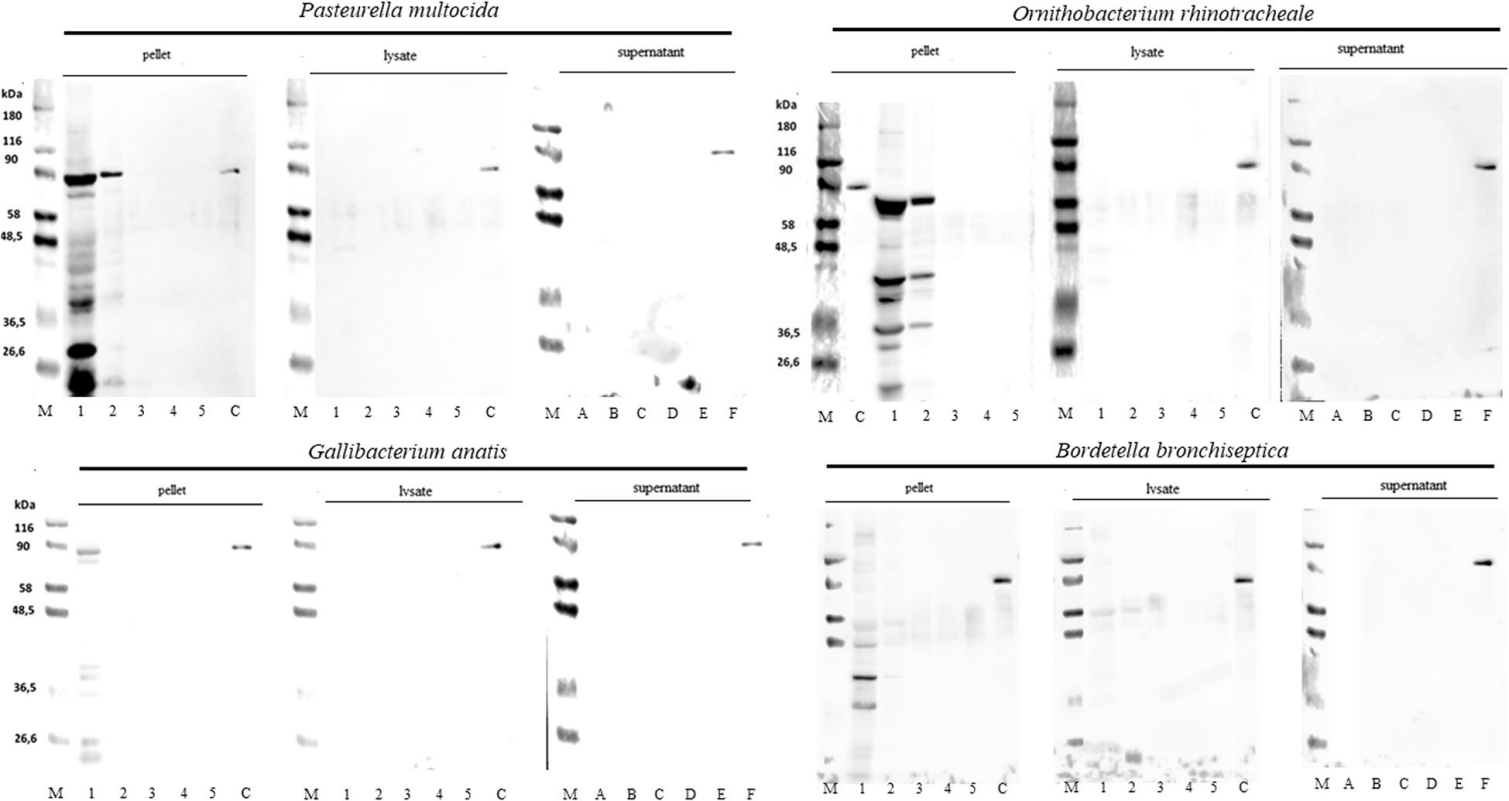
Evaluation of 1G7G8 antibody reactivity against related and non-related bird bacterial cultures in Western blot. Related bird bacteria cultures: *Pasteurella multocida* (FARPER-069), *Ornithobacterium rhinotracheale* (FARPER-172)*,* and *Gallibacterium anatis* (FARPER-173). Non-related bird bacteria culture: *Bordetella bronchiseptica* (FARPER-118). Lane M: Molecular weight marker (26.6–180 kDa). For bacterial lysate and pellet concentrations: lane 1 (500 μg/mL), lane 2 (50 μg/mL), lane 3 (5 μg/mL), lane 4 (0.5 μg mL), lane 5 (0.05 μg/mL), and lane C (recombinant TBDT). For bacterial supernatant concentrations: (**a)** (undiluted supernatant), (**b**) (diluted 1:2), (**c**) (diluted 1:4), (**d**) (diluted 1:8), (**e**) (BHI broth), and (**f**) (recombinant TBDT)

For pellet samples, nonspecific bands were detected at 500 μg/mL for all bacteria, which included a band of ~90 kDa only for *G. anatis*, *P. multocida* and *O. rhinotracheale*. We also detected a specific band of ~90 kDa at 50 μg/mL for *P. multocida*. In addition to a ~90 kDa band, other bands of different molecular weights were also detected for *O. rhinotracheale* at 50 μg/mL. No additional bands were detected for other concentrations (Fig. 3).

### Reactivity of the 1G7G8 monoclonal antibody against *Av. paragallinarum* serogroups in indirect ELISA assay

**Fig. 4:**
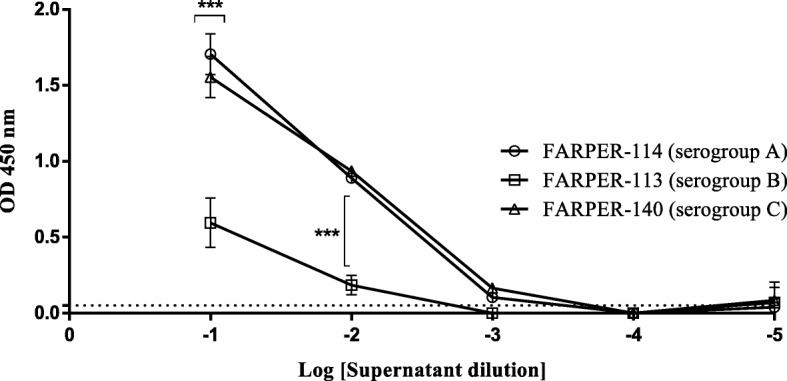
Reactivity evaluation of the 1G7G8 antibody using *Avibacterium paragallinarum* culture supernatants. *Avibacterium paragallinarum* culture: FARPER-113 (serogroup B), FARPER-114 (serogroup A) and FARPER-140 (serogroup C). Error bars represent the standard deviation (SD). The dotted line represents the cutoff that was calculated as the mean + 2SD. ***: *p* = 0.001. A two-way ANOVA and Tukey’s post hoc test were used

**Fig. 5:**
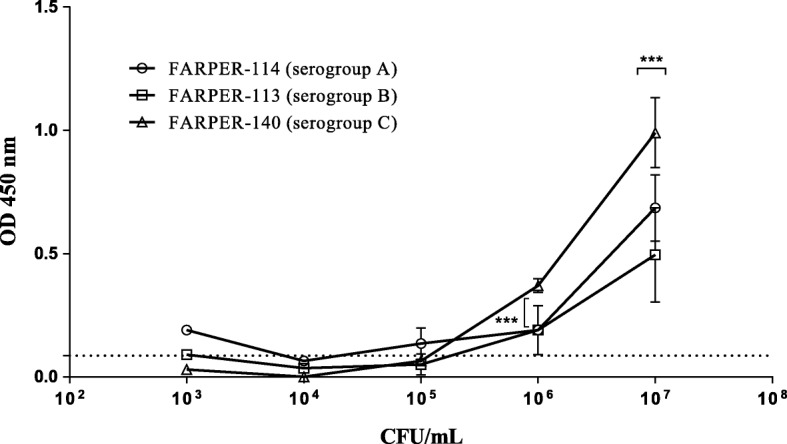
Reactivity evaluation of the 1G7G8 antibody using *Avibacterium paragallinarum* culture lysates. *Avibacterium paragallinarum* culture: FARPER-113 (serogroup B), FARPER-114 (serogroup A) and FARPER-140 (serogroup C). Error bars represent the standard deviation (SD). The dotted line represents the cutoff that was calculated as the mean + 2SD. CFU: colony-forming units. ***: p = 0.001. A two-way ANOVA and Tukey’s post hoc test were used

Reactivity differences between lysates (CFU/mL) and supernatant dilutions from the three *Av. paragallinarum* serogroups were detected. Positive reactivity was observed at 1:10 and 1:100 dilutions of the culture supernatants, and a higher reactivity was observed for serogroups A and C than for serogroup B (*p* = 0.001). Moreover, positive reactivity slightly above the cutoff (0.05) was also detected at a 1:1000 dilution for serogroups A and C (Fig. 4). Regarding lysates, we observed positive reactivity at 10^7^ and 10^6^ CFU/mL for serogroups A, B and C with higher reactivity observed for serogroup C than for the other serogroups (*p* = 0.001). Furthermore, positive reactivity slightly above the cutoff (0.086) was also detected at 1 × 10^5^ CFU/mL for serogroup A (Fig. 5).

### Characterization of the prototype LFT

The assembly of the prototype was performed by Creative Diagnostics (Shirley, NY, USA) using 1G7G8 as a detection and capture antibody (self-pairing). The self-pairing LFT was initially tested using recombinant TBDT protein. The results showed a red line in the T and C line regions, which was considered a positive result (data not shown). All technical details related to prototype assembly are of commercial interest and therefore these cannot be provided.

The assembly prototypes were sent to FARVET Laboratories in Peru and used to evaluate the performance of the LTF using a variety of culture samples. A positive red line was observed in the T line region as well as in the C line region for all three serogroups (serogroup A, B and C) obtained from culture supernatants and lysates of *Av. paragallinarum*. On the other hand, no red line in the T line region was observed for lysates and supernatants from other bacterial cultures such as *G. anatis*, *P. multocida*, *O. rhinotracheale* and *B. bronchiseptica.* In addition, no positive T lines were observed for supernatants obtained from cell cultures infected with infectious laryngotracheitis virus, avian metapneumovirus or Newcastle disease virus (Table 1).

**Table 1:** Specificity evaluation for the prototype LFT using bacterial and viral cultures

Organism	host/cell culture	LFT results	
		Lysates	Supernatants
Bacteria			
*Avibacterium paragallinarum (A)* (FARPER-114)	Chicken	+	+
*Avibacterium paragallinarum (B)* (FARPER-113)	Chicken	+	+
*Avibacterium paragallinarum (C)* (FARPER-140)	Chicken	+	+
*Ornithobacterium rhinotracheale* (FARPER-172) (isolated from Chincha, Ica, Peru)	Chicken	–	–
*Gallibacterium anatis* (FARPER-173) (isolated from Pucallpa, Ucayali, Peru)	Chicken	–	–
*Pasteurella multocida* (FARPER-069) (isolated from Pucallpa, Ucayali, Peru)	Duck	–	–
*Bordetella bronchiseptica* (FARPER-118) (isolated from Cañete, Lima, Peru)	Guinea pig	–	–
Viruses			
Infectious laryngotracheitis virus (VFAR-043)	LMH	N.A	–
Avian metapneumovirus (SHS-FAR)	VERO	N.A	–
Newcastle disease virus (LaSota)	DF-1	N.A	–

+: positive result (T and C red lines); −: negative results (C red line). N.A (Not applicable)

**Fig. 6:**
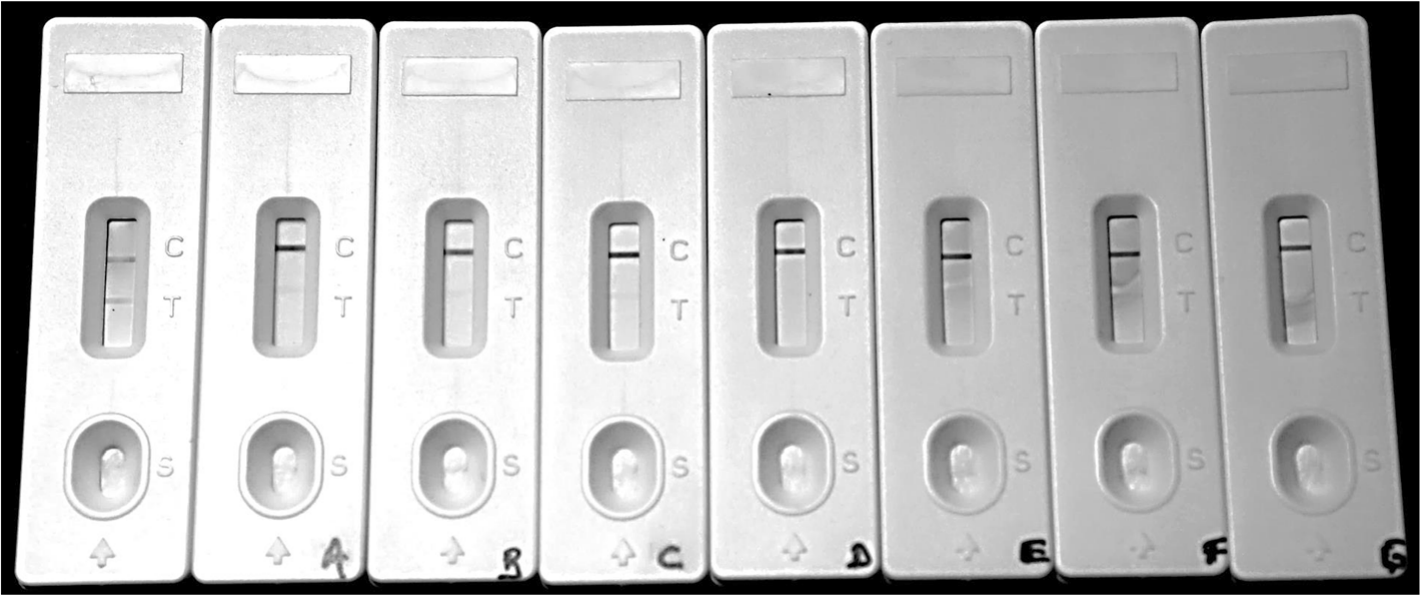
Limit of detection for the prototype LFT. Control (C) line; Test (T) line. *Avibacterium paragallinarum* lysate FARPER-140 (serogroup C) at 10-fold dilution. From left to right: Not labeled test, 10^7^ CFU/mL; (**a**), 10^6^ CFU/mL; (**b)**, 10^5^ CFU/mL; (**c**), 10^4^ CFU/mL; (**d**), 10^3^ CFU/mL; (**e**), 10^2^ CFU/mL; (**f)**, 10^1^ CFU/mL; (**g**), BHI broth. The detection threshold for the LFT was 10^4^ CFU/mL

**Fig. 7:**
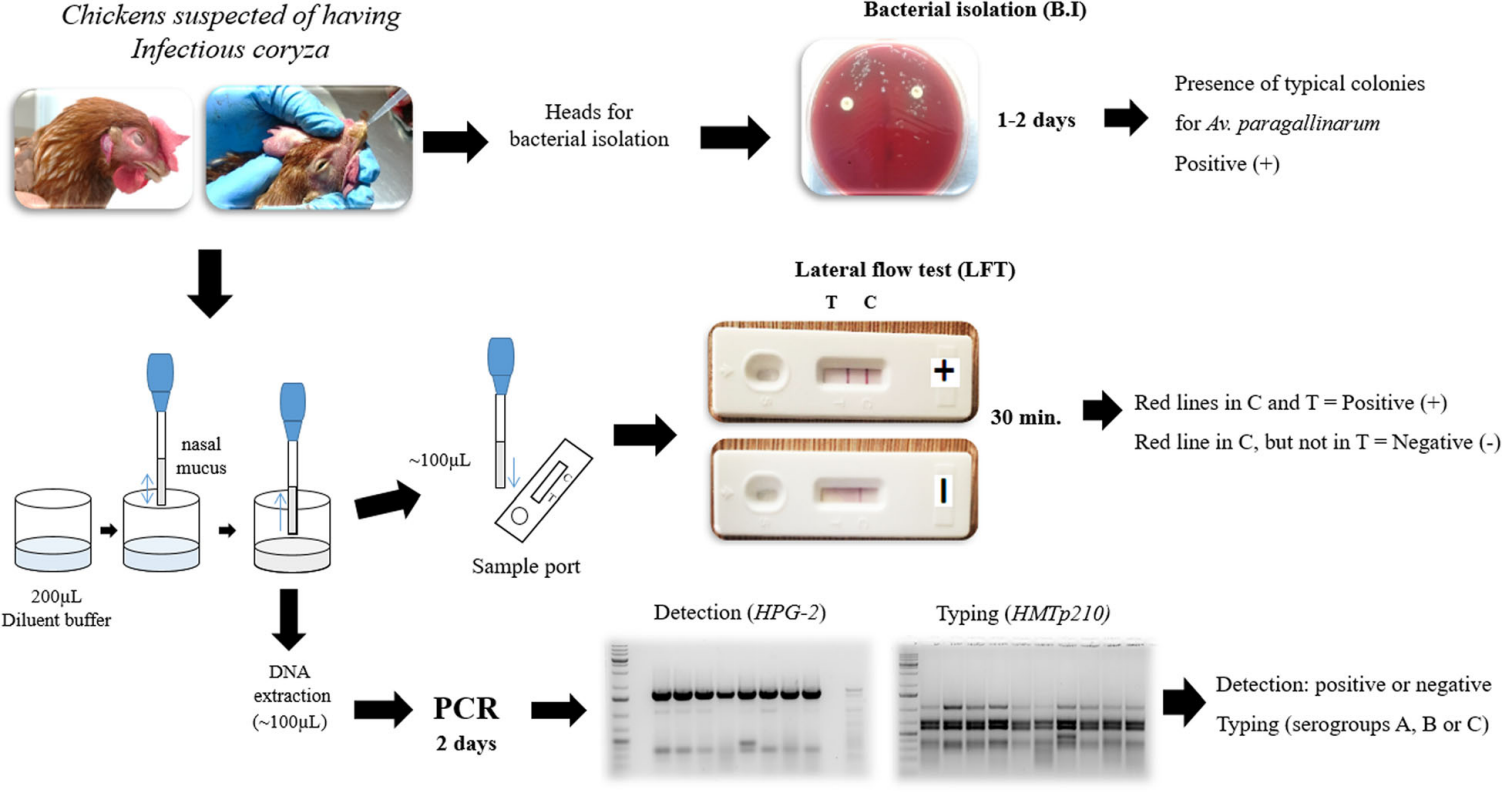
Workflow for evaluation of chickens suspected of having infectious coryza. The evaluation was carried out using bacterial isolation (B.I), polymerase chain reaction (PCR) and lateral flow test (LFT)

The limit of detection was only determined for FARPER-140 (serogroup C) because it had the highest reactivity observed in indirect ELISA assay. The limit of detection was 1 × 10^4^ CFU/mL (Fig. 6). On the other hand, a total of 35 chicken nasal mucus samples were collected from two farms located in the south of Peru in October and December 2017 and in January 2018 (Table 2). To evaluate the specificity and diagnostic sensitivity of the self-pairing prototype LFT, three detection methods, bacterial isolation (B.I), polymerase chain reaction (PCR) and self-pairing LFT, were compared (Fig. 7). The results from the three detection methods are shown in Table 3.

**Table 2:** Detailed description of chickens involved in the study

Farm localization/date	Chicken	Type of chicken	Age of chicken (weeks)	*Coryza vaccine* ^*a*^	Antibiotic treatment^b^	Clinical signs^c^
City of Arequipa						
Suspected farm 1 Oct-2017	A1	Laying hens	23	Yes	No	+
	A2	Laying hens	23	Yes	No	–
	A3	Laying hens	23	Yes	No	+
	A4	Laying hens	23	Yes	No	–
	A5	Laying hens	23	Yes	No	+
	A6	Laying hens	23	Yes	No	+
City of Ica						
Suspected farm 2 Dic-2017	T1	Laying hens	4	Yes	Yes	+
	T2	Laying hens	4	Yes	Yes	+
	T3	Laying hens	4	Yes	Yes	–
	T4	Laying hens	4	Yes	Yes	+
	T5	Laying hens	4	Yes	Yes	–
	T6	Laying hens	4	Yes	Yes	–
	T7	Laying hens	4	Yes	Yes	–
	T8	Laying hens	4	Yes	Yes	–
Suspected farm 3 Jan-2018	I1	Laying hens	7	Yes	No	+
	I2	Laying hens	7	Yes	No	+
	I3	Laying hens	7	Yes	No	+
	I4	Laying hens	7	Yes	No	+
	I5	Laying hens	7	Yes	No	+
	I6	Laying hens	7	Yes	No	+
	I7	Laying hens	7	Yes	No	+
	I8	Laying hens	7	Yes	No	–
	I9	Laying hens	7	Yes	No	–
	I10	Laying hens	7	Yes	No	–
	I11	Laying hens	7	Yes	No	+
	I12	Laying hens	7	Yes	No	+
	I13	Laying hens	7	Yes	No	+
	I14	Laying hens	7	Yes	No	+
	I15	Laying hens	7	Yes	No	+
	I16	Laying hens	7	Yes	No	+
	I17	Laying hens	7	Yes	No	–
	I18	Laying hens	7	Yes	No	+
	I19	Laying hens	7	Yes	No	–
	I20	Laying hens	7	Yes	No	–
	I21	Laying hens	7	Yes	No	–

^a^The vaccination program varies between farms

^b^The antibiotic treatment varies between farms

^c^Clinical signs was judged by veterinary experts. +: positive for clinical signs and -: negative for clinical signs

**Table 3:** Comparative description of *Avibacterium paragallinarum* detection using bacterial isolation (B.I), PCR and the prototype LFT

Chicken	Bacterial isolation			PCR		LFT
	*Avibacterium paragallinarum*	*Gallibacterium anatis*	*Ornithobacterium rinotracheale*	Detection^a^	Typing^b^	
A1	+	+	–	+	B	+
A2	+	–	–	+	B	+
A3	+	–	–	+	B	+
A4	–	–	–	–	–	–
A5	+	–	–	+	B	+
A6	+	–	–	+	B	+
T1	–	–	–	+	B	+
T2	–	–	–	+	A	+
T3	–	–	–	–	–	–
T4	–	–	–	+	A	+
T5	–	–	–	+	A	–
T6	–	–	–	–	–	–
T7	–	–	–	+	B	–
T8	–	–	–	–	–	–
I1	+	+	–	+	B	+
I2	–	+	–	+	B	+
I3	+	+	–	+	B	+
I4	+	+	–	+	B	+
I5	+	+	–	+	B	+
I6	–	–	–	+	B	+
I7	+	+	–	+	B	+
I8	–	–	–	+	B	+
I9	–	+	–	+	B	–
I10	+	+	–	+	B	+
I11	+	+	–	+	B	+
I12	+	–	–	+	B	+
I13	–	–	–	+	B	+
I14	–	–	–	+	B	+
I15	+	–	–	+	B	+
I16	–	–	–	+	B	+
I17	–	–	–	+	B	–
I18	–	+	–	+	B	–
I19	+	–	–	+	B	+
I20	+	–	–	+	B	+
I21	+	–	–	+	B	+

+: positive results; −: negative results. *Avibacterium paragallinarum* serogroups: A, B and C

^a^Target gene *HPG-2*

^b^Target gene *HMTp210*

**Fig. 8:**
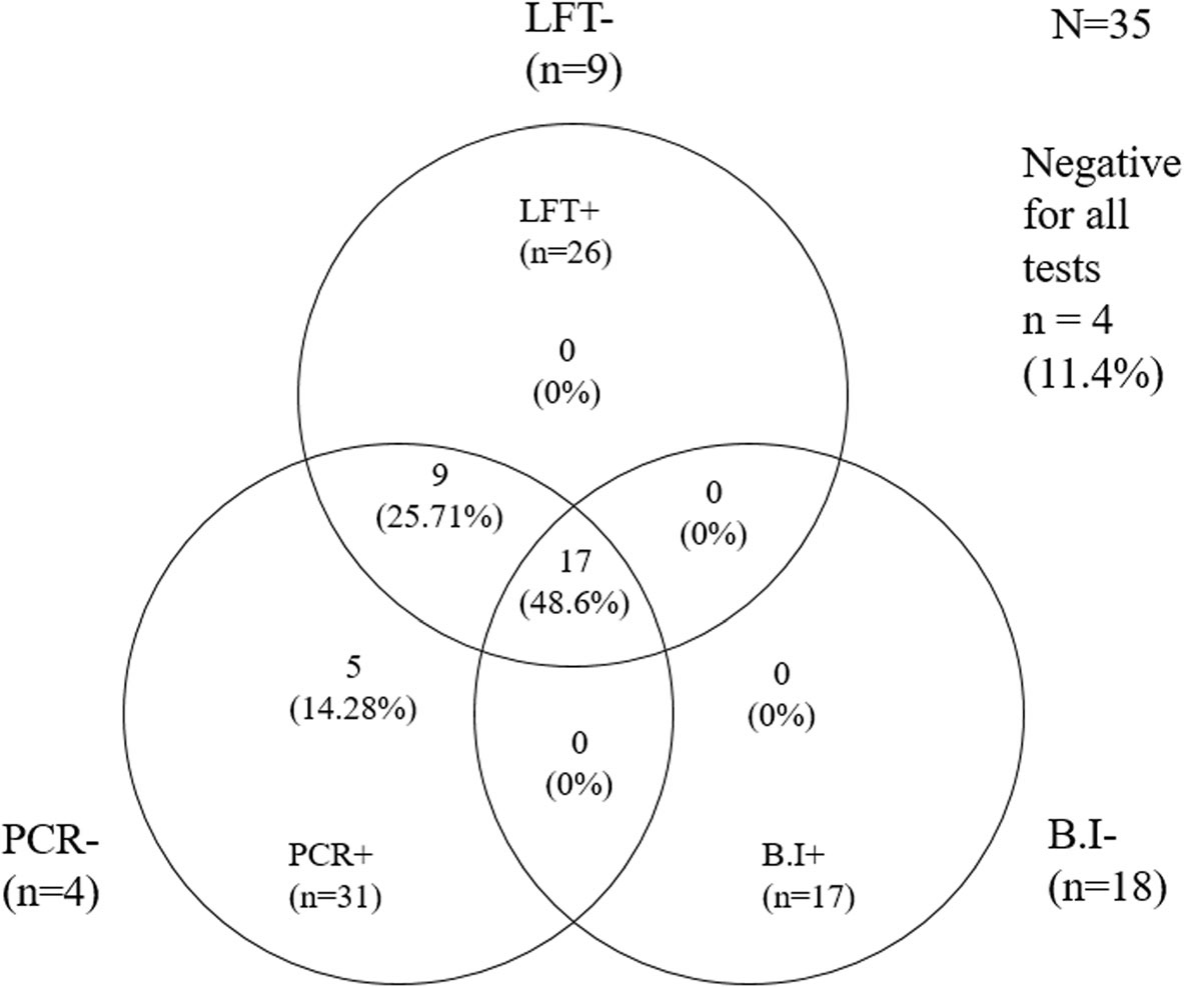
Venn diagrams showing results from the evaluation of chicken nasal mucus samples (*N* = 35). These results were obtained using bacterial isolation (B.I), PCR and the prototype LFT. Samples positive (+) for *Avibacterium paragallinarum* are represented as n (%) inside the diagram, and negative samples (−) are represented as n outside diagram. n = numbers. Areas of overlap indicate positive results in common. Adapted from Stewart et al. BMC Veterinary Research (2017) 13:131

We identified 17 out of 35 clinical samples (51.4%) as positive *Av. paragallinarum* samples by B.I. Additionally, 11 out of 35 samples were positive for *G. anatis* using B.I. However, 88.57% (31/35) of the chicken samples were positive for *Av. paragallinarum* by PCR. From these 31 samples, 28 samples were typed by PCR as serogroup B and 3 samples as serogroup A. The self-pairing prototype LFT gave a positive result for *Av. paragallinarum* in 74.28% (26/35) of the chicken samples. All positive samples from B.I (17/35) were detected by PCR and LFT. Only 11.4% (4/35) samples were negatives from all tests. Nine samples were only positive from LFT and PCR. Additionally, 5 samples were exclusively positive from PCR and negative from B.I and LFT (Fig. 8).

For indicator comparison, self-pairing LFT was compared with B.I and PCR, which were used as reference methods. For comparison between B.I and self-pairing LFT, we obtained 100% Se, 50% Sp, 65.4% PPV, 100% NPV and K of 0.49. This index was considered a moderate agreement between LFT with B.I. For comparison between PCR and self-pairing LFT, we obtained 83.9% Se, 100% Sp, 100% PPV, 44.4% NPV and K of 0.54 (Table 4). This index was considered a moderate agreement between LFT with PCR. Finally, a higher sensitivity was observed when the self-pairing prototype LFT was compared with B.I than when it was compared with PCR (100 and 83.9%, respectively). However, greater specificity was observed when the self-pairing prototype LFT was compared with PCR than when it was compared with B.I (100 and 50.0%, respectively).

**Table 4:** Comparative indicators of the prototype LFT using bacterial isolation (B.I) and PCR

Prototype LFT					
Comparative techniques	Sensitivity	Specificity	PPV^a^	NPV^b^	Kappa index
	% (95% CI)	% (95% CI)	% (95% CI)	% (95% CI)	(95% CI)
B.I	100.0	50.0	65.4	100	0.49
	(81.6–100)	(29.0–71.0)	(46.2–80.6)	(70.1–100.0)	(0.21–0.78)
PCR	83.9	100.0	100.0	44.4	0.54
	(66.4–92.9)	(51.0–100.0)	(87.1–100.0)	(18.9–49.0)	(0.17–0.91)

*95% CI* Confidence interval at 95%

^a^Positive predictive value

^b^Negative predictive value

## Discussion

IC is caused by *Av. paragallinarum*. This disease is diagnosed by clinical signs and confirmed by bacterial isolation [6]. Several factors such the requirement of specific growth media make *Av. paragallinarum* diagnosis a difficult and tedious task [5]. Some molecular techniques such as PCR have been used in order to achieve highly sensitive results [5, 6]. Despite this technique being faster than bacterial isolation, it is still expensive to implement in local laboratories and includes costs transporting samples from farms.

In the present study, we were able to develop a lateral flow test based on the detection of the TonB-dependent transporter (TBDT) through the use of a monoclonal antibody (1G7G8). This antibody was able to detect the expected molecular weight of TBDT (~ 90 kDa) from bacterial cell cultures in Western blot (Fig. 2). Likewise, 1G7G8 allowed us to determine the reactivity differences between *Av. paragallinarum* serogroups using an indirect ELISA assay. Despite all serogroups showing positive reactivity, serogroups A and C had the highest reactivity for supernatants and lysates (Figs. 4 and 5). Therefore, all these results highlighted the ability to use 1G7G8 to detect all three serogroups, which emphasized its use for the development of the lateral flow test (LFT).

The use of the same antibody (self-pairing) as a detection and capture reagent for prototype LFT development has been previously reported for the detection of foot-and-mouth disease virus (FMDV) in cloven-hoofed animals [15]. In this study, the self-pairing prototype LFT allowed us to detect the TonB-dependent transporter from nasal mucus samples from chickens suspected of having infectious coryza.

Nasal mucus discharge is one of the main clinical signs in chickens infected with *Av. paragallinarum* [16]. *Av. paragallinarum* presence in nasal mucus discharge has been detected using molecular techniques such as the TaqMan Real-Time PCR assay [17]. Consequently, we chose to use nasal mucus as a suitable type of sample for lateral flow test evaluation.

The evaluation of thirty-five nasal mucus samples using LFT showed high sensitivity (100%) when it was compared with bacterial isolation (B.I), which indicated that LFT is capable of detecting *Av. paragallinarum*. However, the LFT evaluation showed low specificity (50%) and low positive predictive value (PPV) (65.4%) (Table 4), which could be related either to B.I. limitations in detecting low bacterial load in nasal mucus samples or to overgrowth of fast-growing bacteria [5, 6, 17].

In the same way, we observed high sensitivity (83.9%) and high specificity (100%) when LFA were compared with PCR, but a low negative predictive value (NPV) (44.4%) was observed (Table 4). This low NPV is due to discrepancies when both methods classify samples as negative. For instance, five samples were negative by LFA and positive by PCR. These discrepancies could be explained by either low bacterial load beneath the limit of detection of LFT or bacterial DNA remnants after antibiotic treatment.

According to the Venn diagram (Fig. 8), we observed that 17 out of 35 evaluated samples were positive using all three methods of detection, and nine samples were positive using only LFT and PCR. Despite 8 out of these 9 positive samples from LFT and PCR showing clinical signs, B.I was not able to detect *Av. paragallinarum* in them. This result indicated that LFT is a more reliable and sensitive method of detection than B.I, which could explain the low specificity (50%) observed when LFT was compared with B.I (Table 4).

On the other hand, despite 4 out of 35 samples being negative for all three methods of detection, PCR was the only method that was able to detect a high portion of positive samples (31/35). This difference with LFT (26/35 positive samples) could explain the obtained sensitivity (83.9%) (Fig. 8 and Table 4). The high proportion of positive samples detected by PCR could have arisen from free DNA remnants of non-viable bacteria resulting from antibiotic treatment, which could be a reason for unnecessary and expensive prevention strategy implementation. Therefore, all these results indicated that LFT is a reliable and trustworthy method of detection that can be used in field conditions.

Previous studies reported coinfection cases of *Av. paragallinarum* with *G. anatis* or *O. rhinotracheale* in chickens [16, 18]. In the present study, we identified *G. anatis* in nasal mucus samples (11/35) by B.I., but only 8 out of these 11 samples were identified as coinfection cases by B.I. These 8 samples were also positive for *Av. paragallinarum* using LFT (Table 3), which supported the capability of LFT to target the protein of interest.

Particular attention should be paid to the fact that nasal mucus samples, which were typed as serogroup B (Table 3), could be detected by LFT despite the fact that bacterial culture samples from serogroup B were the least reactive by indirect ELISA assay (Figs. 4 and 5). This result emphasizes the ability of LFT to detect all three serogroups. On the other hand, recent studies indicated the poor performance of mPCR [19, 20], which was used in this study for sample typing. Nonetheless, the conventional serotyping technique of hemagglutination inhibition (HI) has also been reported to have sensitivity limitations and to be a laborious technique [21]. Moreover, HI is a technique that depends on bacterial isolation, and in the present study, only 51.4% of all samples were successfully isolated using B.I. Therefore, in order to type the largest number of samples, we used an alternative and sensitive technique based on PCR assay, which is still being used to type field samples [6]. Interestingly, we could detect the serogroups A and B in the same farm (Table 3). This event is supported by a coinfection case of two serogroups A and B reported previously in other geographic region [22]. These two independent cases indicate the need for future assessments in order to understand the dynamic of *Av. paragallinarum* serogroups in a given farm.

Likewise, the limit of detection of the present LFT was 1 × 10^4^ CFU/mL, which is similar to other lateral flow tests previously reported. Thus, a limit of detection of 1 × 10^4^ CFU/mL and 1.8 × 10^5^ CFU/mL was found for *Salmonella sp.* in chickens [23] and for *Escherichia coli* O157:H7 in bovine and swine animals [24], respectively. This comparative description underscores that the LFT developed in the present study has a similar limit of detection to that reported in other studies.

Finally, one of the two limitations of this study was the lack of specificity evaluation using other *Avibacterium spp.* (*Av. avium*, *Av. endocarditis*, *Av. gallinarum* and *Av. volantium*), and the other one was related to the number of cultures of positive isolates from *Av. paragallinarum*. Therefore, these two limitations should be taken into consideration for further validation assays of this prototype.

## Conclusion

A prototype self-pairing LFT for *Av. paragallinarum* detection was successfully developed in this study. The use of this prototype in the field does not require specially trained personnel, and this test evaluation can be performed in 30 min. In light of our results, the prototype self-pairing LFT has potential applications for rapid detection of *Av. paragallinarum* in chickens suspected of having infectious coryza in field. However, further validation assays are required using reference and field strains from different laboratories in other geographical regions.

## Methods

### Bioinformatics prediction

Using as reference reported sequences of TBDT in *Av. paragallinarum* (GenBank: WP_052716793.1, KKA99715.1, WP_051185305.1 and WP_017806794.1), we annotated the TBDT gene (GenBank: MG242130.1) on the previously reported genome of *Av. paragallinarum* strain 72 [25]. The following regions were flagged on the protein sequence: the signal peptide (predicted using SignalP 4.1) and regions with an identity of 50% or higher with any protein of other avian pathogens (identified using BLASTp). Then, peptides were predicted on the unflagged regions using Bebipred 2.0 and Optimum Antigen (GenScript Laboratories, Piscataway, NJ, USA), selecting those peptides with the highest conservation and located in the outer part of the protein structure (by analyzing the 3D model). The best candidate obtained (named Ma-4) was used in the downstream analysis.

### Monoclonal antibody production using a peptide

For monoclonal antibody production, the peptide (Ma-4) was synthesized and used to immunize a BalbC mouse which was provided by GenScript Laboratories. After 5 weeks, spleen cells were isolated for hybridoma production by fusion with the SP2/0 mouse myeloma line. Peptide-reactive clones were finally selected by ELISA assay and then isotyped. Antibody titers in hybridoma supernatants were calculated as the highest positive dilution against the peptide by indirect ELISA assay. The peptide synthesis, hybridoma production, clone selection, isotyping and antibody titers were performed by GenScript Laboratories. The positive clones were sent to FARVET Laboratories for monoclonal antibody production according to a previously published methodology [26]. The hybridoma culture supernatants were used for the antibody purification steps.

The mouse was euthanized using cervical dislocation without anesthesia following the American Veterinary Medical Association (AVMA) guidelines and the terminated animal was disposed by contracted biological waste disposal company [27].

### Antibody purification

Chromatographic runs were conducted on a commercial prepacked affinity HiTrap rProtein A FF column (0.7 cm × 2.5 cm) (GE Healthcare, Uppsala, Sweden), which occurred at room temperature, and an AKTA Pure system (GE Healthcare). The column was equilibrated with 8 column volumes (CV) of 200 mM sodium phosphate buffer and 3 M NaCl at pH 7. The hybridoma culture supernatants were previously centrifuged for 10 min at 4 °C at 13000 x g, filtered using a 0.22 μm filter and then loaded into the column at 156 cm/h linear flow velocity using a 150 mL superloop (GE Healthcare). After washing unbound compounds with 8 CV of the equilibration buffer, the antibodies were eluted stepwise with 8 CV of 0.1 M sodium citrate at pH 3.5. Column eluates were continuously collected as 1 mL fractions in a F9-R fraction collector (GE Healthcare).

### Preparation of recombinant protein

The sequence of the gene encoding the TonB-dependent transporter (TBDT) was obtained from NCBI reference sequence MG242130. The protein sequence consists of 756 amino acids with a theoretical molecular weight of 86.5 kDa. The expression of this recombinant protein was carried out in an *E. coli* BL21 strain, and a 6x-histidine tag was added at the amino terminus (Creative Diagnostics, Shirley, NY, USA). This recombinant protein was produced to be used as a positive control in Western blots.

### Bacterial pellet, lysate and culture supernatant

The *Av. paragallinarum* isolates FARPER-113 (serovar B-1), FARPER-114 (serovar A-2) and FARPER-140 (serovar C-1) from an outbreak in Peru were serotyped (Page and Kume scheme), characterized and grown using methods that were previously reported [2]. The bacteria were grown in brain-heart infusion (BHI) broth (Sigma Aldrich Co., St Louis, Missouri, USA) supplemented with 5% equine serum and 0.01% NAD (TM/SN) for 18 h under agitation at 37 °C [2]. Later, the *Av. paragallinarum* cultures were centrifuged at 11000 x g for 5 min at 4 °C. The supernatants were filtered using a microfilter (0.45 μm) and stored at − 80 °C until use. The bacterial pellet was resuspended with 10 mL of phosphate-buffered saline, pH 7.4, plus Tween 20 5% (*v*/v) (Sigma Aldrich Co.), and incubated on ice for 30 min. Subsequently, the resuspended bacterial pellet was sonicated at an amplitude of 100 (On/Off time, 30 s/10 s) for 2 min on ice using a sonicator S-4000 (Qsonica, LLC, Newton, CT, USA) and centrifuged at 11000 x g for 30 min at 4 °C. Finally, the bacterial lysates obtained from sonication were stored at − 80 °C, the pellet was resuspended using 1 mL of 8 M urea (Sigma Aldrich Co.) and was stored at − 20 °C. Culture lysates and pellets were quantified using a Bradford assay (Merck KGaA, Darmstadt, Germany). Additionally, the pellets, lysates and supernatants of related bird bacteria cultures, such as *Ornithobacterium rhinotracheale* (FARPER-172), *Gallibacterium anatis* (FARPER-173), and *Pasteurella multocida* (FARPER-069) and non-related bird bacteria *Bortedella bronchiseptica* (FARPER-118), were locally isolated and cultivated using standard bacteriological techniques [16, 28–30]. For a better understanding, the serovars B-1, A-2 and C-1 obtained using Page and Kume scheme were referred as serogroups B, A and C, respectively.

### Identification of TBDT protein using Western blot

Bacterial lysate, pellet and culture supernatant samples were separated by 4–20% sodium dodecyl sulfate-polyacrylamide gel electrophoresis (SDS-PAGE) and transferred to a nitrocellulose membrane using an e-blot device (GenScript Laboratories). The membranes were blocked with Azure Chemi Blot clocking buffer (Azure Biosystems, Dublin, CA, USA) with shaking for 1 h at room temperature. Membranes were washed three times for 10 min with phosphate-buffered saline (PBS)-Tween 20 and were incubated with purified monoclonal antibodies (0.4 μg/mL). Membranes were washed three times with phosphate-buffered saline-Tween 20. The immune reaction was performed with peroxidase-labelled goat IgG anti-mouse antibody (GenScript Laboratories) using radiance as a substrate and revealed by a CCD camera (Azure Biosystems). For bacterial lysates and pellets, 10-fold dilutions were prepared with the previously quantified samples (500, 50, 5, 0.5, 0.05 and 0.005 μg/mL). For bacterial culture supernatants, 2-fold dilutions were prepared with the supernatant (1:2, 1:4, 1:8 and 1:16) in PBS at pH 7.0. For recombinant TBDT protein, 500 ng was used. For molecular weight estimation of the studied proteins, we used a pre-stained molecular weight marker that ranged from 26,600 to 180,000 Da (Sigma Aldrich Co.) and a molecular weight marker that ranged from 30,000 to 120,000 Da (GenScript Laboratories).

### Reactivity of monoclonal antibodies using indirect enzyme-linked immunosorbent assay (ELISA)

The 96-well microplate (Greiner Bio-One GmbH, Kremsmünster, Austria) was coated with 100 μL of antigen. The antigens used were bacterial lysates (10^3^–10^7^ CFU/mL) that were diluted with buffer carbonate/bicarbonate, pH 9.5, and supernatants from bacterial culture (dilutions from 1:10 to 1:100,000). All antigens except bacterial lysates were diluted in PBS at pH 7.4. Microplates were incubated at 4 °C overnight. Microplates were washed once with 150 μL of wash buffer (PBS + 0.05% Tween 20), blocked for 1 h at room temperature with 100 μL of blocking buffer (PBS + 0.5% BSA) and washed three times with 150 μL of washing buffer. One-hundred microliters purified monoclonal antibodies (5 μg/mL) were added to the plates and incubated for 1 h at 37 °C. Microplates were washed three times with 150 μL of washing buffer. Then, 100 μL of horseradish peroxidase conjugated goat anti-mouse IgG H&L (IgG-HRP) (Abcam, Cambridge, MA, USA) was added, and the microplates were incubated for 1 h at 37 °C. The microplates were washed four times using 150 μL of washing buffer; then, 100 μL of 3,3′,5,5′-tetramethylbenzidine (TMB) ELISA substrate (high-sensitivity) (Abcam) was added, and the microplates were incubated at room temperature for 30 min. Finally, the reaction was stopped using 100 μL of 2 N H_2_SO_4_, and the microplates were read at 450 nm using an EON microplate reader instrument (Biotek, Winooski, VT, USA).

### Assembly of a prototype lateral flow test (LFT)

All possible combinations of antibodies were evaluated in order to determine which antibody pair was the most suitable for test development. All antibodies were tested as capture and detection reagents. Thus, all antibodies were conjugated to gold nanoparticles and used for the test. The detection antibody was immobilized at the Test (T) line region, and an anti-mouse IgG antibody was immobilized at the Control (C) line region of a nitrocellulose membrane. Briefly, two red lines in C and T regions meant a positive result, and only one red line in the C region meant a negative result for *Av. paragallinarum*. No band in the C region meant an invalid LFT.

### Specificity evaluation of the prototype LFT

Evaluation of specificity was carried out using culture lysates and supernatants of related and non-related bird bacterial cultures that were prepared as described above. All culture lysates were diluted until reaching 1 × 10^7^ CFU/mL, from which 100 μL was mixed with 100 μL of diluent buffer. Finally, 100 μL was added to the sample port of the LFT.

Additionally, cell cultures were infected with avian respiratory viruses in order to evaluate LFT specificity. Cell lines such as chicken hepatocellular carcinoma cell line (LMH), chicken fibroblast cell line (DF-1) and African green monkey kidney cell line (VERO) were seeded at 1 × 10^6^ cells/mL with Dulbecco’s modified Eagle medium (DMEM) F12 (HyClone, Logan, UT, USA) supplemented with 5% heat-inactivated fetal bovine serum (FBS) in a 75 cm^2^ flask and cultivated in 5% CO_2_ at 37 °C until reaching 70% confluency. These cell lines were infected with infectious laryngotracheitis virus (VFAR-043 strain) [31], which was initially adapted to the LMH cell culture following a previously reported method [32], Newcastle disease virus (LaSota strain) [33] or avian metapneumovirus (SHS-FAR strain) [34] at 0.02 multiplicity of infection (MOI). The infected cells were cultivated until a cytopathic effect was observed. Then, the supernatants were collected and centrifuged at 3000 g for 5 min at room temperature. Finally, the supernatants with viruses were quantified using plate assay and expressed in plaque-forming units (PFU)/mL. The quantified supernatants were diluted until reaching 1 × 10^6^ PFU/mL, and 100 μL was mixed with 100 μL of diluent buffer and added to the sample port.

### Limit of detection of the prototype LFT

The limit of detection was performed using *Av. paragallinarum* culture lysates from FARPER-140 (serogroup C). Colony count was performed following a previously reported method [17]. A bacterial culture (50 mL) was quantified (1 × 10^6^ CFU/mL) and centrifuged at 11000 x g for 5 min at 4 °C. The bacterial pellet was resuspended with 5 mL of phosphate-buffered saline, pH 7.4, plus 5% Tween 20 (*v*/v) (Sigma Aldrich Co.), incubated on ice for 30 min and sonicated based on factors mentioned above in order to obtain 1 × 10^7^ CFU/mL. The lysates were serially diluted at 10-fold from 1 × 10^7^ to 1 × 10^1^ CFU/mL in diluent buffer. The lowest positive dilution was considered the limit of detection of the test.

### Chicken sample evaluation using the prototype LFT, PCR and bacterial isolation

Thirty-five chickens suspected of having infectious coryza were sent from farms located in the south of Peru (cities: Arequipa and Ica) to FARVET laboratories, after 2 days of clinical sign onset, for routine evaluations. Chicken nasal mucus was obtained by exerting pressure on the nasal sinuses. The nasal mucus was taken using a 2-mL transfer pipette and mixed with 200 μL of diluent buffer in a 1.5-mL microtube until reaching a homogeneous mixture. From this mixture, 100 μL was taken to perform the LFT, and 100 μL was used for the PCR assays. The homogeneous mixture was added to the sample port of the LFT, and the results were assessed visually after 30 min. PCR assays based on the *HPG-2* gene [35] were used for detection, and a multiplex PCR (mPCR) based on the *HMTp210* gene [21] was only used for chicken nasal mucus typing. Bacterial isolation was performed using bird heads according to a previous report [35].

All chickens were euthanized using cervical dislocation without anesthesia following the American Veterinary Medical Association (AVMA) guidelines [36] and the disposal of dead animals was performed according to the Peruvian Regulation [37].

### Statistical analysis

All quantitative data were analyzed using GraphPad Prism 6.0 (GraphPad Software, La Jolla, CA, USA). Indirect ELISA assays performed for duplicate and optical density (O.D.) values were represented as the mean and standard deviation (SD). The cutoff was calculated as the mean O.D. value +2SD. The O.D. value used for cutoff calculation was obtained from the 1:10,000 dilution for supernatants and 1 × 10^4^ CFU/mL for lysates. The O.D. values obtained above the cutoff were considered positive, and those below the cutoff were negative. Two-way ANOVAs with Tukey’s post hoc test were used for serogroup comparison analysis (*p* < 0.05).

Two-by-two contingency tables were created to analyze associated values among the three methods of detection. The following indicators for the prototype LFT were calculated using bacterial isolation and PCR as reference methods: sensitivity (Se), specificity (Sp), positive predictive value (PPV), negative predictive value (NPV) and kappa index (K). Each indicator was calculated using a 95% confidence interval. Kappa index (agreement level) was denoted using Landis and Koch values [38], where 0 is poor, 0.01–0.2 is slight, 0.21–0.4 is fair, 0.41–0.6 is moderate, 0.61–0.8 is substantial and 0.81–1.0 is almost perfect.

## Abbreviations

*Av. paragallinarum*: *Avibacterium paragallinarum*
*B. bronchiseptica*: *Bortedella bronchiseptica*
B.I: Bacterial isolation
BHI: Brain-heart infusión
BSA: Bovine serum albumin
CFU: Colony-forming unit
CV: Column volumes
DF-1: Chicken fibroblast cell line
DMEN: Dulbecco’s modified Eagle medium
ELISA: Enzyme-Linked ImmunoSorbent Assay
FBS: Fetal bovine serum
FMDV: Foot-and-mouth disease virus
*G. anatis*: *Gallibacterium anatis*
HI: Hemagglutination inhibition
IC: Infectious coryza
IgG-HRP: Horseradish peroxidase conjugated goat anti-mouse IgG H&L
K: Kappa index
kDa: Kilo dalton
LFT: Lateral flow test
LMH: Chicken hepatocellular carcinoma cell line
mPCR: Multiplex Polymerase Chain Reaction
NAD (TM/SN): Complete growth medium that contains both chicken serum and nicotinamide adenine dinucleotide
NCBI: National Center for Biotechnology Information
NPV: Negative predictive value
*O. rhinotracheale*: *Ornithobacterium rhinotracheale*
O.D: Optical density
OMVs: Outer membrane vesicles
*P. multocida*: *Pasteurella multocida*
PBS: Phosphate-buffered saline
PCR: Polymerase chain Reaction
PFU: Plaque-forming unit
PPV: Positive predictive value
SD: Standart desviation
SDS-PAGE: Sodium dodecyl sulfate–polyacrylamide gel electrophoresis
Se: Sensitivity
Sp: Specificity
TBDTs: TonB-dependent transporters
TMB: 3,3′,5,5′-tetramethylbenzidine
VERO: African green monkey kidney cell line
